# Cell‐free production of pore forming toxins: Functional analysis of thermostable direct hemolysin from *Vibrio parahaemolyticus*

**DOI:** 10.1002/elsc.201600259

**Published:** 2017-12-11

**Authors:** Srujan Kumar Dondapati, Doreen A Wüstenhagen, Eckhard Strauch, Stefan Kubick

**Affiliations:** ^1^ Fraunhofer Institute for Cell Therapy and Immunology (IZI) Branch Bioanalytics and Bioprocesses Potsdam‐Golm (IZI‐BB) Potsdam Germany; ^2^ Federal Institute for Risk Assessment Department of Biological Safety National Reference Laboratory for Monitoring Bacteriological Contamination of Bivalve Molluscs Berlin Germany

**Keywords:** Lipid bilayer, Pore forming, TDH, Toxin, *Vibrio parahaemolyticus*

## Abstract

The pore forming characteristic of TDH1 and TDH2 variants of thermostable direct hemolysin (TDH), a major toxin involved in the pathogenesis of *Vibrio parahaemolyticus*, was studied on a planar lipid bilayer painted over individual picoliter cavities containing microelectrodes assembled in a multiarray. Both proteins formed pores upon insertion into the lipid bilayer which was shown as a shift in the conductance from the baseline current. TDH2 protein was able to produce stable currents and the currents were influenced by external factors like concentration, type of salt and voltage. The pore currents were influenced and showed a detectable response in the presence of polymers which makes them suitable for biotechnology applications.

**Abbreviations**
TDH: thermostable direct hemolysin
TM: translation mixture

## 1 Introduction

Thermostable direct hemolysin (TDH) is a proteinaceous toxin considered to be a major virulence factor associated with *Vibrio parahaemolyticus* strains ability to cause food borne gastroenteritis 1, 2. TDH is a protein of 165 amino acid residues with a single intramolecular disulfide bond near the carboxy terminus 3. Apart from the monomer configuration, TDH is one of the few pore‐forming toxins which exist as an oligomer in solution without any association with the membrane unlike other pore‐forming toxins. It was shown that TDH forms a tetramer in solution and possesses strong hemolytic activity 4, 5. The crystal structure of the TDH tetramer was demonstrated by electron microscopy and molecular simulation studies showing a central pore with dimensions of 23 Å in diameter and ∼50 Å in depth 4, 5. By using single amino acid substitutions of residues involved in π‐cation interaction, it was shown that tetramer configuration is correlated to the hemolytic activity of the protein 5. TDH binds to the erythrocyte membrane and forms pores of around 2 nm on the surface resulting in the colloid osmotic lysis. This membrane pore allows both chloride ions activated by calcium and water to pass through the membrane, resulting in an altered ion flux leading to diarrhea during infection 6. On Wagatsuma blood agar plates TDH exhibits beta‐hemolysis which is widely referred as Kanagawa phenomenon (KP) 7. Apart from hemolysis, TDH performs a variety of biological activities, including cytotoxicity, cardiotoxicity, and enterotoxicity 8, 9. KP positive bacterial strains contain one or two TDH gene copies 10. In total, five different TDH gene variants have been described which share a sequence identity >96.7% 11. Apart from KP effect, TDH is known to exhibit the so called Arrhenius effect, where the hemolytic activity is inactivated when the protein is heated at 60°C and is reactivated by further heating at 90°C for 15 min 12. This effect is observed in several bacterial toxins including α‐hemolysin of *Staphylococcus aureus*.

In humans, infections by *Vibrio parahaemolyticus* are caused by strains of different serotypes. The serogroup O3:K6 revealed to have pandemic potential as strains of this serogroup have spread throughout the world within a decade 13. Also some cases in Europe were reported recently 14. In O3:K6 pandemic strains, two TDH genes termed TDH1 and TDH2, are present and are responsible for the Kanagawa phenomenon. The gene TDH2 holds 97.2% homology with TDH1 and was found primarily responsible for the phenotypic expression of hemolytic activity 11. Very recently, these bacterial strains were reported to exhibit antibiotic resistance 14. In this context, it is of highest importance to elucidate the mechanism of pore formation by TDH. Studying the pore forming functionality of toxins on lipid bilayers is widely used for understanding the mechanism of the toxins such as α‐hemolysin of *Staphylococcus aureus*, Eltor hemolysin from *Vibrio cholera*, aerolysin from *Aeromonas hydrophilia*, VacA protein from *Helicobacter pylori* and anthrax toxin from *Bacillus anthracis* 15, 16, 17. Although there were some preliminary reports about the single channel activity of TDH, it was demonstrated without any specificity to a particular variant and the properties were also demonstrated with a focus on mutant toxicity 18, 19. Moreover the reconstitution of the protein was shown in the presence of planar bilayers derived from unsaturated lipids and higher percentage of cholesterol.

In order to understand the exact mechanism of TDH induced hemolysis, it is important to investigate how TDH incorporates into the cell‐membrane and forms pores. This is possible by overexpressing the protein and further purification of the desired protein. Whereas cell‐based expression of the toxins could cause toxic effects to the host cells, cell‐free protein synthesis systems are becoming an efficient alternative for the synthesis of cytotoxic proteins 20, 21. In these open cell‐free systems, one can easily modify the reaction components according to the required conditions to achieve optimum function for the protein of interest. Cell‐free protein synthesis is an extremely fast procedure and one can assess the functionality of the protein immediately without any further tedious and time consuming purification steps. By carefully designing the plasmid or a linear PCR product with the addition of all essential regulatory elements necessary for cell‐free protein synthesis, one can produce a wide range of proteins ranging from soluble proteins to transmembraneous proteins, antibody fragments etc 19, 22, 23. Thus cell‐free protein synthesis is an ideal method for the production of toxins, prior to their functional characterization. Recently different variants of TDH including TDH1 and TDH2 were synthesized by an *E.coli* based cell‐free expression. Functionality was demonstrated on blood agar plates 24. Based on these findings we chose two proteins TDH1 and TDH2 and further exploited the single‐channel properties of these proteins in order to understand the pore forming mechanism and their biological transport properties.

In this report, we reconstituted two individual proteins, TDH1 and TDH2, produced in a cell free system, into planar lipid bilayers painted over microelectrode arrays using the Orbit16 system [Nanion GmbH; Munich] 25, 26, 27. The main objective of our work is to synthesize and demonstrate systematically the pore forming activity of the protein directly from the cell‐free reaction mixture without any further purification. Additionally, we analyzed preliminarily the transport activity of the protein for PEG polymers of different size in order to explore the nanopore properties of this protein for analytical sensing.

## 2 Materials and methods

### 2.1 Cell‐free synthesis of thermostable direct hemolysin

Genes encoding TDH1 and TDH2 were obtained by gene synthesis (GeneArt Gene Synthesis, LifeTechnologies). Sequence data of both the genes were presented in the supplementary information. Both TDH1 and TDH 2 were synthesized in an *E.coli* based cell‐free system 21, 24. Total reaction was around 50 μL with 35% (v/v) of the reaction mixture was *E. coli* lysate containing T7 RNA‐polymerase, 40% of the reaction mixture containing complete aminoacids (1.25 mM each) and 25% containing plasmid (5 nM), 14C leucine (25 μM of 100 dpm/mol) and water. Coupled transcription‐translation reactions were performed in a thermomixer (37°C, 500 rpm) for 90 min, followed by qualitative and quantitative analysis of 14C labeled samples (100 dpm). For toxin detection and functional assessment we used non‐radioactive samples.

### 2.2 SDS–PAGE and autoradiography

Qualitative information of the protein was obtained by running SDS–PAGE electrophoresis of the translation mixture after precipitation with cold acetone as described 20, 24. First, 5 μL of the translation mixture was added to an Eppendorf tube. To this, 45 μL of Millipore water was added followed by the addition of 150 μL of cold acetone. After incubating the Eppendorf tubes on ice for 15 min, samples were centrifuged for 10 min at 16 000 g at 4°C. Later the supernatant was removed completely and the pellet was dried by incubating at 37°C for 15 min. Next, 20 μL of loading buffer was added to the tubes and mixed by vortexing. Finally, samples were loaded onto a precast NuPAGE 10% Bis‐Tris gel for SDS‐PAGE analysis. After electrophoresis, the gels were stained with coomasie blue and dried. Next the dried gels were analyzed by autoradiography (Typhoon Trio, GE Healthcare Life Sciences).

### 2.3 Protein quantification

Protein quantification was done by hot trichloroacetic acid (TCA) precipitation as described 20, 24. In brief, 5 μL of the translation mixture was added to a 10 mL test tube. To this, 3 mL of 5% TCA solution containing 2% (w/v) casein acid hydrolysate was added and the samples were incubated at 80°C for 15 min. Immediately, reaction test tubes were placed on ice and incubated for 30 min to precipitate the synthesized protein. After this step, the solution was filtered on a glass microfiber filter (filtration paper MNGF‐3 Macherey‐Nagel) followed by a washing step with TCA. Next the filter papers were dried with acetone. Later the dried filter papers were placed in a scintillation vial and filled with an appropriate volume of scintillation liquid (Quicksafe A, Zinsser Analytic). After incubation at room temperature for 1 h, samples were counted in a liquid scintillation counter (Beckman Coulter, Inc.).

### 2.4 Lipid bilayer formation and electrophysiology recordings

Lipid bilayers were formed from 1,2‐diphytanoyl‐sn‐glycero‐3‐phosphocholine (DPhPC), Cholesterol (CHO) (Avanti Polar Lipids, Albaster, AL, USA). Lipids were dissolved in octane (Sigma Aldrich, Munich, Germany) in a ratio 80:20 (DPhPC:CHO) at a concentration of 2 mg/mL. Concentrations of 200 mM, 500 mM or 3M KCl (Sigma Aldrich (Fluka), Munich, Germany), 10 mM Hepes, buffered at pH 7.0 was used as an electrolyte. Monodispersed poly (ethylene glycol) (monoPEG‐3000, Mw = 2700–3300 g/mol) and Polydispersed poly (ethylene glycol) (polyPEG‐1500, Mw = 1400‐1600 g/mol) (FLUKA, Sigma Aldrich, Munich, Germany) were dissolved in the electrolyte solution to a concentration of 10 mg/mL and 2 μL of this solution was added to the 200 μL electrolyte in the measurement chamber for recordings.

Figure 2 shows the scheme of functional characterization of the TDH on a planar lipid bilayer formed over a 50 μm cavity on a 16 microelectrode array. The cavity contains the nonpolarizable working electrode containing Ag/AgCl layer deposited on the underlying Cr/Au layer as described in 26. Briefly, 200 μL of electrolyte solution was added to the measurement chamber of an Orbit 16 System (Nanion Technologies GmbH, Munich, Germany). Bilayer formation was performed as described 25, 27. For the automated bilayer formation on the 16 cavities in parallel, a small amount (approx. 0.3 μL of DPhPC: CHO (80:20) at 2 mg/mL in octane was applied to the chip surface beside the sub‐picoliter cavities containing individual microelectrodes 25, 27. Subsequently, the counter magnet was turned repeatedly (up to 10 times) at a speed of 45–180°/s to evenly distribute the lipid‐solvent mixture over the surface, leading to an increasing fraction of the cavities being electrically sealed. Once the lipid bilayer was formed, the seal resistance jumps from megaohms to gigaohms and the lipid bilayer then separates the cavity containing the working electrode from the external solution. After confirming the lipid bilayer presence, around 2 μL of TDH sample (2.6 μg/mL final concentration) was added into 200 μL buffer solution containing the reference electrode, and waited for the reconstitution. Different holding potentials were applied to the underlying bilayer to characterize the pore formation. Once the pore was inserted, we have added the polymers and PEG molecules to the solution containing the reference electrode.

A single channel amplifier (EPC‐10, HEKA Electronic Dr. Schulze GmbH, Lambrecht, Germany) was connected to the multiplexer electronics port of the Orbit16 system. Recordings were done at a sampling rate of 50 kHz with a 10 kHz Bessel filter. Data were analyzed with Clampfit (Molecular Devices, Sunnyvale, CA, USA).

## 3 Results and discussion

### 3.1 Cell‐free protein synthesis and quantification

Once the translation reaction was completed, the reaction mixture was analyzed for the presence of de novo synthesized 14C labeled proteins. The pattern of proteins was analyzed on SDS‐PAGE electrophoresis and autoradiography from the whole translation mixture (TM) and also from the supernatant (SN) fraction. Active protein was also isolated by using his‐tag purification and all the fractions collected during the purification protocol were analyzed by SDS‐PAGE and quantified as explained in methods.

**Figure 1:**
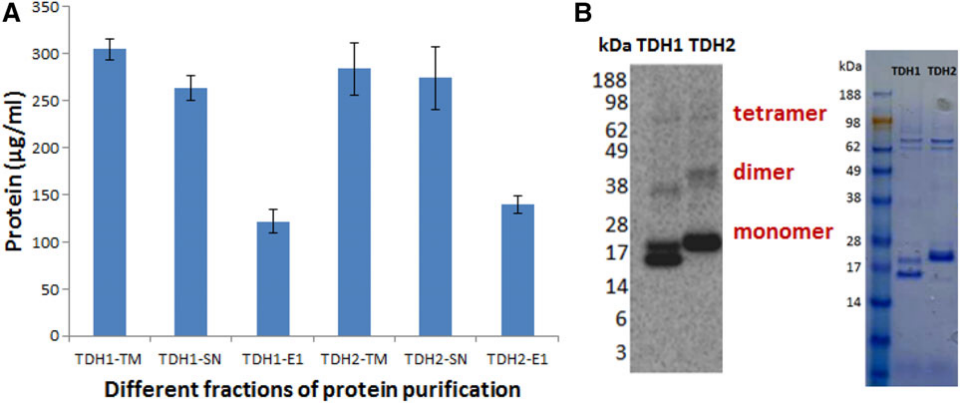
Synthesis of TDH1 and TDH2 in the *E.coli* based cell‐free system. (A) Protein quantification (TM: translation mixture, SN: supernatant, E1: first elution fraction from his tag purification). Protein quantification was performed by hot trichloroacetic acid (TCA) precipitation as described 20, 24. (B) Protein analysis by SDS‐PAGE electrophoresis: Autoradiography showing the bands corresponding to TDH1 and TDH2 (left) and Coomasie stained gel showing the bands from the elution fractions of TDH1 and TDH2 (right).

Figure 1 shows both the quantitative and qualitative analysis of TDH1 and TDH2 synthesized in the *E.coli* derived cell‐free system. Figure 1A shows the results of both TDH1 and TDH2 protein yields at different steps of purification. Both the proteins were synthesized quite efficiently and most of the protein was present in the SN fraction. SN fraction was used for further purification by his‐tag procedure and also for functional studies. In both cases, elution fraction obtained during the his‐tag purification retained 40–60% of the total synthesized protein, which is a sufficient amount of protein for functional measurements. Figure 1B shows the assembly of both the proteins after his‐tag purification. Multiple bands at approximately around 20, 40, and 80 kDa on the gel were observed which could be obtained from the oligomeric configurations of the protein (Fig. 1B). It was shown in the literature that TDH exhibits several configurations in the solution ranging from widely reported homodimer to tetramer without any membrane support unlike other pore forming toxins 5. Both proteins were identified by the presence of a single band at around 20 kDa before his‐tag purification (data not shown).

SDS‐PAGE electrophoresis without any radioactive label showed a thick monomer band at around 20 kDa in the elution fraction of the his‐tag purification (Fig. 1B). By combining observations from both the SDS‐PAGE and autoradiography, one can clearly conclude that both the TDH variants were successfully produced and purified in the cell‐free system.

### 3.2 Pore formation by TDH

**Figure 2:**
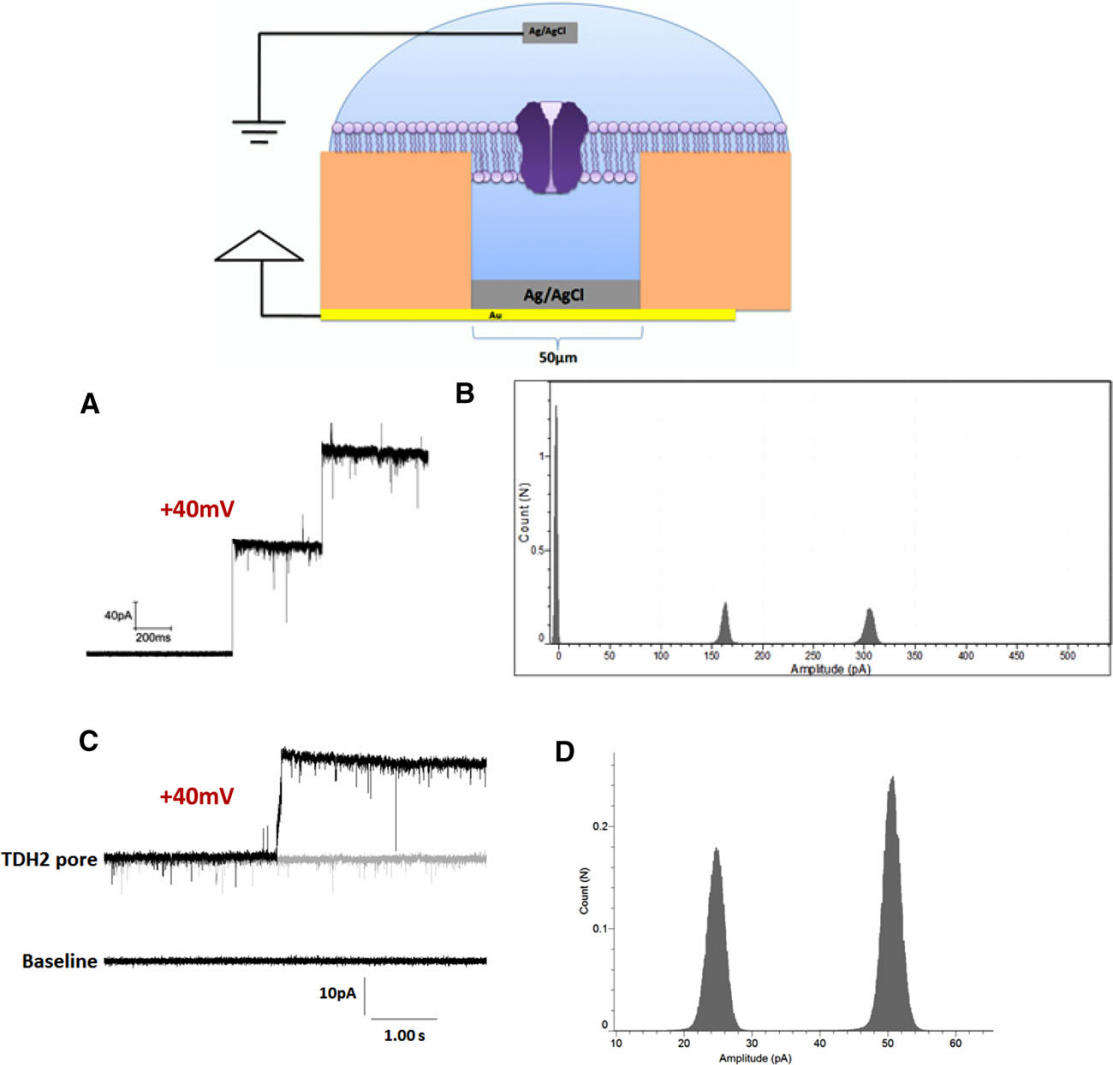
Insertion of TDH1 and TDH2 proteins into the lipid bilayer formed from DPhPC: CHO over a 50 μm cavity (V_applied_ = 40 mV) at room temperature. (A) Current measurements following the addition of TDH1 into the solution (3 M KCl, 10 mM Hepes, pH 7.0) surrounding the membrane show the stepwise incorporation of two proteins. (B) Histogram data of A) showing the 1 nS conductance for each protein insertion (C) Current measurements following addition of TDH2 into the solution (200 mM KCl, 10 mM Hepes, pH 7.0) surrounding the membrane, shows the stepwise incorporation of two proteins. (D) Histogram data of (C) showing the same 0.62 nS conductance for each protein insertion (V_applied_ = +40 mV).

For pore formation, we used SN fraction for all our experiments presented in this paper. Figure 2A shows the pore insertion of TDH1 in a step wise manner at a holding potential of +40 mV. Two pores are inserted corresponding to the two current levels. The plotted histograms show the individual current peaks corresponding to the pore insertion (Fig. 2B). All the measurements related to TDH1 were done in the presence of 3M KCl, 10 mM Hepes, pH 7.0 solution. At low salt concentrations, there were no clearly defined pore currents and moreover the pore response is not stable for long term characterization. Although the TDH1 induces the individual transmembrane pore currents, the protein suffers from the long term stability problems unlike TDH2. In Fig. 2C, currents obtained due to the insertion of TDH2 were presented. At an applied voltage of +40 mV, first pore insertion created a current jump up to 24 pA followed by the second channel of equal conductance. The histogram shows independent peaks at 24 and 50 pA corresponding to two TDH2 insertions (Fig. 2D). The TDH2 pore is quite stable when compared to TDH1 and was thus further used for long term characterization of their properties. A total of eight control measurements were made by using cell‐free reaction samples prepared without the addition of plasmid. Although there were some noisy currents which disrupted the lipid bilayer, there was never a stable pore formation observed in any of the control measurements.

### 3.3 Effect of voltage on the TDH2 transmembrane currents

Effect of voltages on the transmembrane currents induced by TDH2 was studied in the presence of NaCl and KCl for TDH2 protein to understand the influence of cation on the protein function. Voltages +40 mV, +60 mV, and +100 mV were applied to the underlying lipid bilayer. Pore currents were plotted against potentials for both salt concentrations. All measurements were performed with symmetrical salt solutions on both sides of the lipid bilayer.

**Figure 3:**
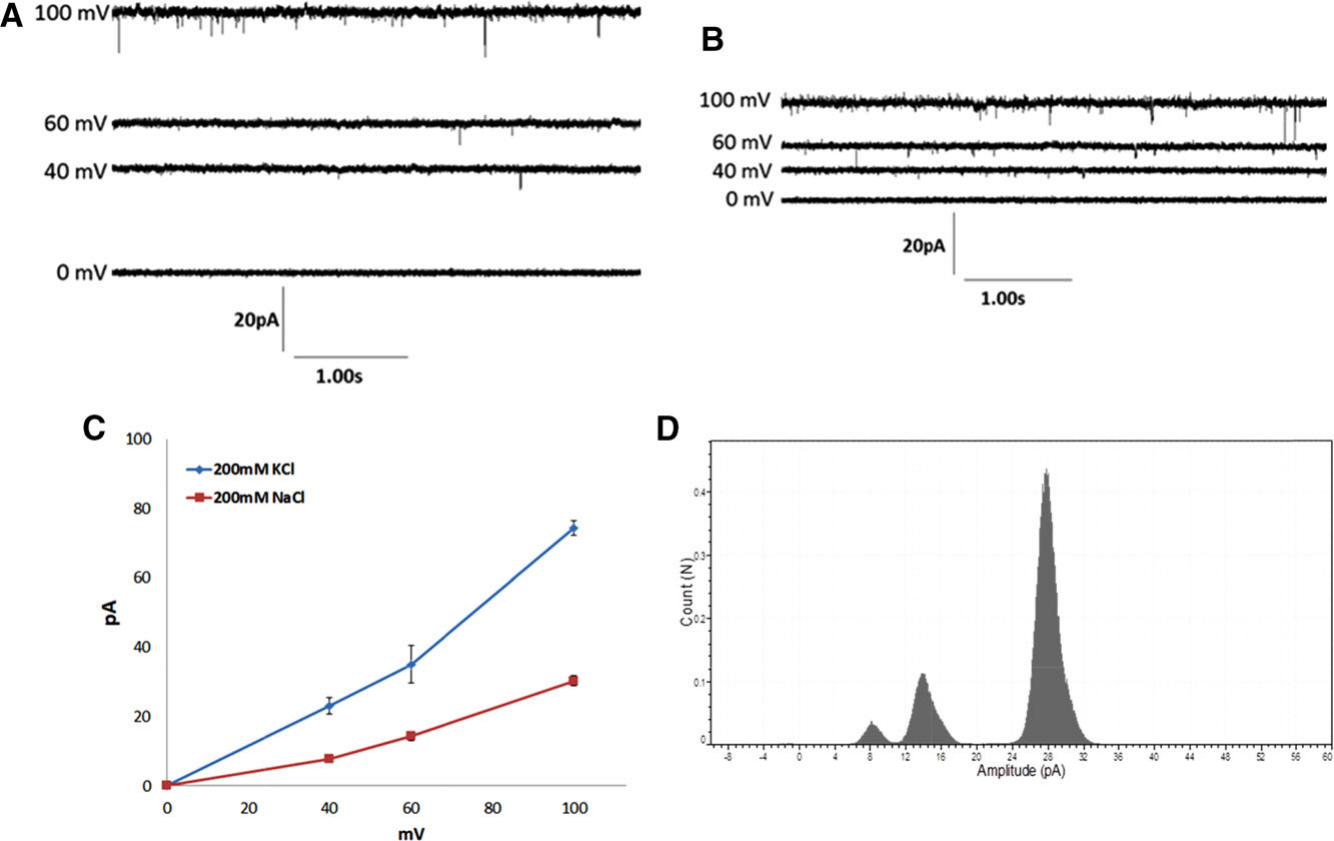
Influence of the type of cation on the transmembrane currents induced by TDH2 at different voltages. (A) Transmembrane currents induced by TDH2 at different voltages in the presence of KCl solution (10 mM Hepes, 200 mM KCl pH 7.0). (B) Transmembrane currents induced by TDH2 at different voltages in the presence of NaCl solution (10 mM Hepes, 200 mM NaCl pH 7.0). (C) Current versus voltage plot of TDH2 recordings from (A) and (B). (D) Histogram data of (B) showing the 0.2 nS conductance of the protein in the presence of NaCl.

Figure 3 shows the influence of cations on the pore conductance of TDH2. In the presence of 200 mM KCl, TDH2 forms pores and currents increase with potential from +40 mV to +100 mV (Fig. 3A). At higher voltages, there were current spikes in the downward direction when compared to lower voltages. Figure 3B shows the influence of voltages on the pore response in the presence of 200 mM NaCl. The influence of voltage on the pore forming behavior in the case of NaCl is the same as KCl. When currents were plotted against voltages, we noticed that the currents increase linearly with voltage in both the cases (Fig. 3C). The pore currents were much larger in the case of KCl when compared to NaCl. This clearly shows that KCl produces a larger response when compared to NaCl. Figure 3D shows the histogram of the pore currents for NaCl at different voltages. Individual peaks in the histogram correspond to currents obtained at +40, +60 and +100 mV. From the experiments described above, we can conclude that KCl is a better electrolyte for transporter function of TDH2. The currents were quite large even at +40 mV in the case of KCl when compared to the +100 mV in the presence of NaCl. This experiment demonstrates that TDH2 has a higher conductance in the presence of potassium ions over sodium ions. In this report our observations were limited to cation selectivity. In future experiments, the influence of anions on the transmembrane current induced by TDH2 at different voltages will be studied to analyze whether TDH2 has a preferential selectivity of anions over cations. Preferential selectivity of potassium over sodium by TDH2 induced pores supports the previous hypothesis 28. In their study over the human erythrocyte membranes, Huntley et al has showed that TDH induced hemolysis was highly selective for potassium ions over sodium. This is a very important observation in the context of TDH induced cationic fluxes playing a major role in the diarrhetic action of *Vibrio parahaemolyticus*. Our observations with the cationic selective pore response clearly match to the erythrocyte model studies 28.

**Figure 4:**
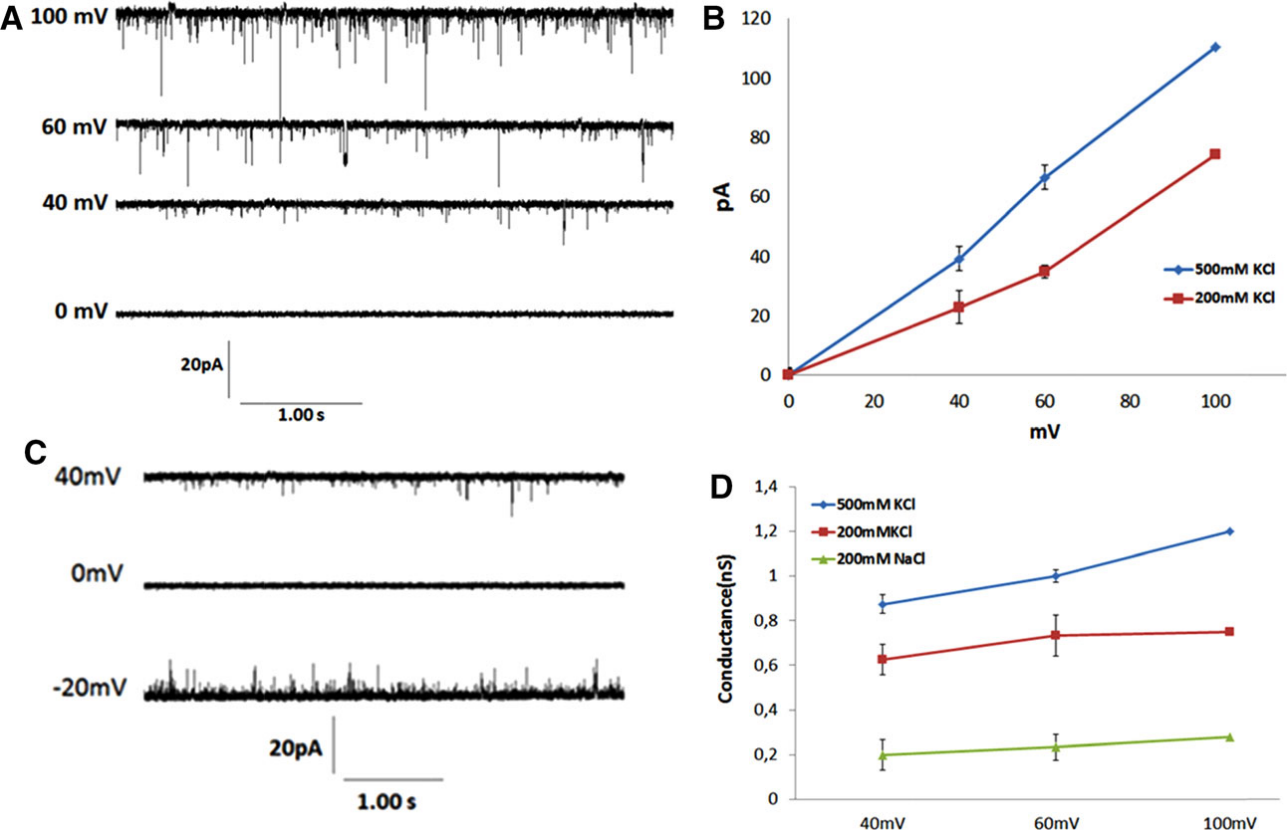
Influence of the salt concentration on the transmembrane current induced by TDH2 at different voltages. (A) Transmembrane currents induced by TDH in the presence of KCl solution (10 mM Hepes, 500 mM KCl pH 7.0). B) Current versus voltage plot of TDH2 recordings from two different salt concentrations. (C) Transmembrane currents induced by TDH2 in the presence of two voltages with opposite polarity (+40 mV and ‐20 mV). (D) Conductance values in different salt concentrations at different voltages (40, 60, and 100 mV).

With increase in the salt concentration from 200 mM to 500 mM KCl, there was an enhancement of pore forming currents of TDH2. Figure 4A shows the currents obtained at 500 mM KCl, 10 mM Hepes, and pH 7.0. We observed that the influence of voltage on the transmembrane currents is similar to the lower salt concentrations as shown in Fig. 3 but the amplitude of the currents was much higher (Fig. 4B). This is a particularly important observation and plays a significant role while working with analytical applications where the sensitivity of analyte detection could be enhanced with a higher signal‐to‐noise ratio. The effect of salt on the nanopore currents has been explained in literature 29. Nevertheless, the long term stability of TDH2 at much higher salt concentrations is important and needs to be studied. We also observed that at negative voltages of −20 mV, the pore currents were much higher when compared to the currents at +40 mV (Fig. 4C). With further increase in the negative voltages the currents were much noisy and the bilayer was broken. Consistently for most of our experiments we chose to work with positive voltages as they are quite stable even at higher voltages of around +100 mV. Conductance values at different voltages in different salt conditions were plotted in Fig. 4D.

### 3.4 Analytical characterization of TDH

Once the pore forming was characterized, we tried to work on the transport properties of the protein. As a consequence it will be significant to analyze the transport of analytes. We first analyzed the transport of polymer molecules through the TDH2 pores. Using PEGs with two different molecular weights 1500 and 3000, we probed the conductance behavior of the TDH2 pore. We added 2 μL of PEG mixtures (10 mg/mL) into the electrolyte solution. Both PEGs individually modified the TDH2 pore currents in a totally different manner.

**Figure 5:**
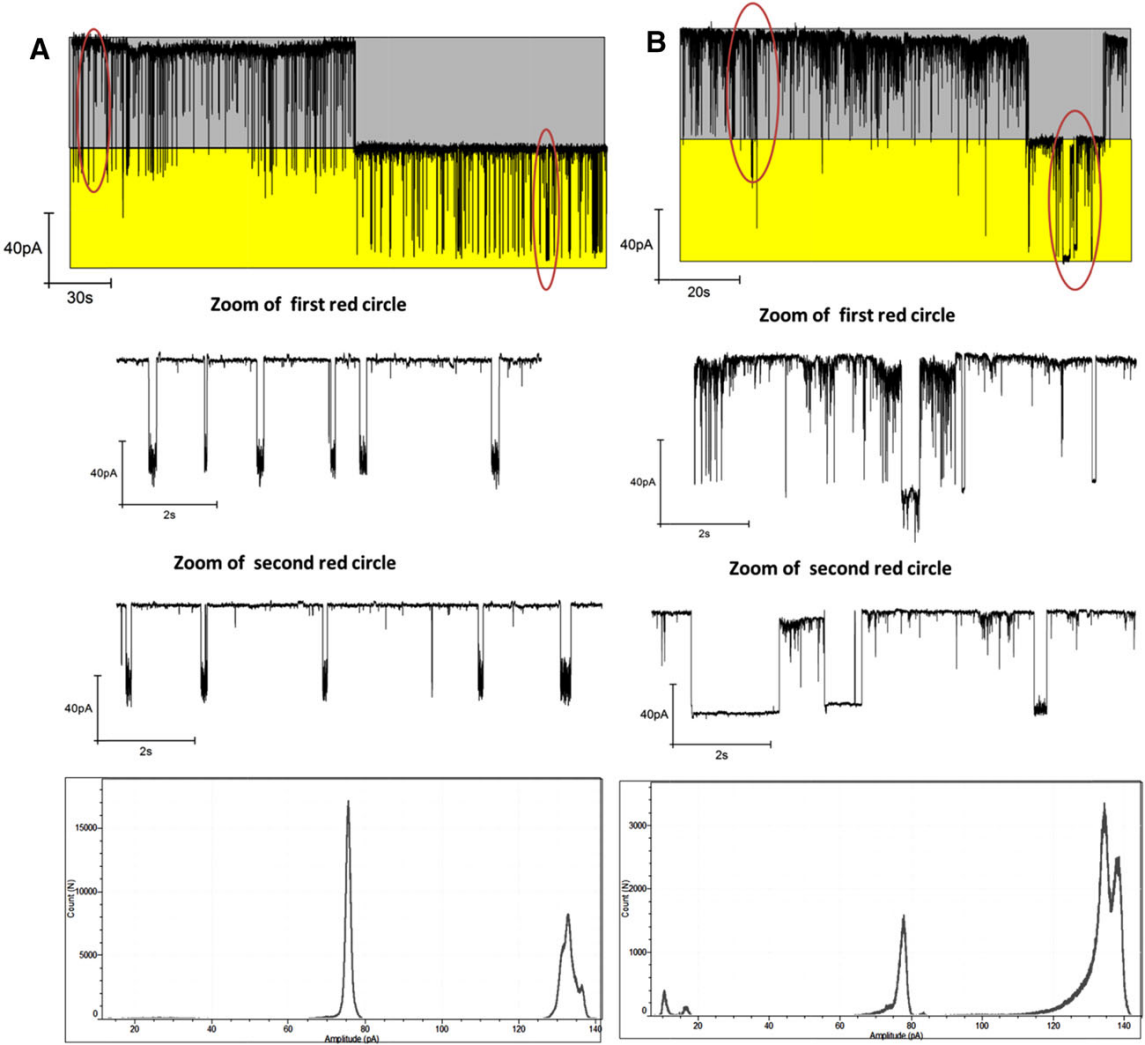
Response of TDH pores in the presence of PEGs. (A) Response of TDH2 in the presence of polymers PEG‐1500 (V_applied_ = +100 mV) (upper). Zoomed out part (from the two red circles) of the main current trace was shown as the top and bottom pictures and histogram of the primary current trace. (B) Response of TDH2 in the presence of PEG‐3000 (V_applied_ = +100 mV) (upper). Zoomed out part (from the two red circles) of the main current trace was shown as the top and bottom pictures and histogram of the primary current trace. In both the cases, PEG molecules reversibly partitions into and out of the pore causing well‐defined blockades. All measurements of TDH2 were done in the presence of 500 mM KCl, 10 mM Hepes, and pH 7.0 buffer.

As observed in Fig. 5A, in the presence of PEG‐1500, TDH2 open pore current showed downward spikes with a 50% conductance level from the fully open state (presented in the colors). After measuring for a while, the conductance of the open pore shifted to 50% (presented in the yellow color) with continuous conductance spikes. When we zoom in a part of current trace (marked by a red circle), we can notice well‐defined current blockade events originated from the pore opening currents which could be due to the transport of PEG‐1500 molecules. A histogram plotted out of the currents measured showed three individual peaks corresponding to the pore currents with open conductance level of around 140 pA and partially closed half conductance level of around 80 pA and a completely blocked conductance level of around 25 pA. When similar experiments were conducted in the presence of high molecular weight PEG‐3000, the TDH2 pore conductance was modified with downward current fluctuations (Fig. 5B). The pore currents showed a conductance shift in between fully open state and half of the open state with continuous downward spikes. When we zoom in a part of the current trace (marked by a red circle), we noticed a rectangular shaped current blockade lasting for milliseconds and then shifted back to the open pore current level which is due to the transport of PEG‐3000 molecules. Histogram plotted out of the currents measured showed three individual peaks corresponding to the pore currents with open conductance of around 140 pA and partially closed half conductance level of around 80 pA and a completely blocked third conductance level of around 20 pA. These observations demonstrate that TDH2 pores can differentiate polymers with different sizes which make them suitable for analytical applications 25, 30. From Fig. 5, we can observe that the blockade events were quite faster and frequent in the case of PEG‐1500 with a short duration time when compared to PEG‐3000. In the case of PEG‐3000, the blockade events are considerably larger and less frequent with a larger duration time. All the recordings in this report were measured in a more general way with regular repainting the broken bilayers in between the measurements in the presence of polymers. This could shift the orientation of the protein as well as the kinetics of the PEG blocking. A more detailed study of polymer partitioning could be done by adding the polymers from different sides (cis and trans) of the lipid bilayer in order to clearly understand the functional mechanism of the TDH2 pore and its shape and size 31.

Based on our experimental results, the pore may have an analytical value after careful characterization and experimental testing.

## 4 Concluding remarks

Two variants of the thermostable direct hemolysin family TDH1 and TDH2 were synthesized in a cell‐free system and purified by the his‐tag method. Both proteins showed good expression levels and formed multimer configurations within the solution. When inserted into planar bilayers over 16 microelectrode cavity array chips, both toxin variants showed pore forming currents. In comparison to TDH1, TDH2 formed stable pores lasting for a long term measurement which makes it suitable for analyte measurements. Several factors like the type of cation, salt concentration and voltage, influence the pore formation of TDH2. The pore conductance of TDH2 is cation dependent and showed larger currents in the presence of KCl when compared to NaCl. The protein displayed an active response in the presence of analytes like polymers which could help for further analysis of the stochastic sensing properties of the protein. The pores were able to differentiate the PEG molecules with two different molecular weights. Thus these proteins reconstituted on microelectrode arrays could serve as biosensing devices in future. Although most of our findings presented here are from a single cavity, parallel recordings of pore conductance properties of the protein from 16 cavities will translate into rapid acquisition of large amounts of uniformly high quality data and thus recording times could be reduced. One of the most important conclusions of our work is to detect the pore response of the synthesized protein directly from the cell‐free mixture without any further purification which makes the cell‐free system a better choice for the fast and efficient synthesis of functional toxins. The pore forming properties from the cell‐free reaction mixture showed a similar response as the his‐tag purified protein. This poreforming property can also be used as an alternative method to the conventional methods for the detection of TDH proteins. As these proteins also form a nanopore, these proteins can be added to a poreforming class of proteins. Additionally, a better understanding of the pore forming characterization of TDH proteins and the pore blocking interaction could also promote the development and production of a new class of antibiotics directed against these proteins. Overall, our findings offer an insight into the mechanism of toxicity of TDH.

*The authors have declared no conflict of interest*.
